# Identifying transcription factors controlling the basal expression of human MRP4 highlights a substantial role for Sp1

**DOI:** 10.1002/2211-5463.70245

**Published:** 2026-04-07

**Authors:** Debora Singer, Angelique Kragl, Katrin Ziems, Juliane Glaubitz, Sophie Grammbauer, Susanne Hildebrandt, Mladen V. Tzvetkov, Gabriele Jedlitschky

**Affiliations:** ^1^ Department of General Pharmacology, Center of Drug Absorption and Transport (C_DAT) University Medicine Greifswald Greifswald Germany; ^2^ ZIK plasmatis Leibniz Institute for Plasma Science and Technology (INP), Greifswald Germany

**Keywords:** *ABCC4/MRP4* gene regulation, chromatin immunoprecipitation, electrophoretic mobility shift assay, Ets transcription factors, Sp1

## Abstract

The multidrug resistance protein 4 (MRP4/ABCC4) is a versatile efflux pump, known to transport several drugs but also signaling molecules such as cyclic nucleotides and lipid mediators. Based on this substrate spectrum and its broad tissue distribution, MRP4 plays a significant physiological and pathophysiological role in both the cardiovascular and oncological fields. However, the determinants of its gene expression are still incompletely defined. This study aimed to identify key regulatory elements and transcription factors that are essential for basal *MRP4* expression. Using luciferase reporter assays with a series of 5′‐deletion constructs, we identified a region upstream of the transcription start site as crucial for basal expression across diverse cell types. This region is evolutionary highly conserved and contains putative binding sites for Sp1 and Ets transcription factors. Site‐directed mutagenesis of both binding elements resulted in a significant decrease in the promoter activity in HeLa and megakaryoblastic M07e cells. The binding of Sp1 to this region was further confirmed by electrophoretic mobility shift and chromatin immunoprecipitation assays. Finally, siRNA knockdown of Sp1 led to a significant decrease in MRP4 protein levels and function. In summary, we show that Sp1 binds to the *MRP4* promoter and plays an essential role in the basal expression of *MRP4*, with Ets factors also potentially cooperating in this regulation.

## Graphical abstract

The MRP4 transporter exports several drugs and signaling molecules. Here, we identified key promoter elements regulating basal *MRP4* expression. Using reporter assays, we defined a conserved region with essential Sp1 and contributory Ets sites, which controlled basal *MRP4* expression. These results were confirmed by mobility shift and chromatin immunoprecipitation assays, while Sp1 knockdown experiments showed reduced MRP4 expression and function.

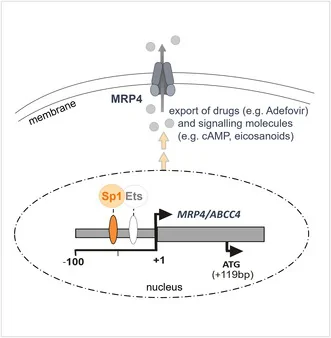

**Abbreviations**
ABCC4/MRP4*: ATP‐binding cassette subfamily C member 4/multidrug resistance protein 4 (Note: *ABCC4* is the gene name according to the HUGO Gene Nomenclature Committee; however, for clarity, the term *MRP4*/MRP4 is used here for gene and protein)
AP2: activator protein 2
bis‐POM‐[^3^H]PMEA: bis(pivaloyloxymethyl)‐[^3^H]PMEA
CAR: constitutive androstane receptor
ChIP: chromatin immunoprecipitation
Elk: Ets‐like
EMSA: electrophoretic mobility shift assay
EPAC: exchange protein directly activated by cAMP
Ets: E26 transformation‐specific
MAPK: mitogen‐activated protein kinase
MEK: mitogen‐activated protein kinase kinase
MYC: myelocytomatosis oncogene
Nrf2: NF‐E2‐related factor 2
PBS: phosphate‐buffered saline
PCV: packed cell volume
PGE2: prostaglandin E2
PKA: protein kinase A
PMEA: 9‐(2‐phosphonylmethoxyethyl)adenine
PPAR: peroxisome proliferator‐activated receptor
S1P: sphingosine‐1‐phosphate
Sp1: specificity protein 1
TBX22: T‐box transcription factor 22

MRP4 (ABCC4)*, a member of the MRP/CFTR subfamily (C‐branch) of the ATP‐binding cassette (ABC) transporters, represents a very versatile export pump for amphiphilic anions. It is expressed in many tissues, with high levels in blood cells, particularly platelets [1, 2, 3]. Its substrate spectrum covers several drugs including nucleoside‐based antiviral compounds and cytostatic drugs such as methotrexate, and importantly, also several endogenous signaling molecules. These include the cyclic nucleotides cAMP and cGMP [2, 4]. MRP4 has been shown to be an independent regulator of intracellular cAMP levels and therefore to be involved in processes such as the proliferation of vascular smooth muscle cells, vasodilatation, and cardiac hypertrophy [5, 6, 7]. In turn, cAMP can autoregulate its own homeostasis by stimulating *MRP4* expression through the exchange proteins activated by cAMP (EPAC) pathway [8, 9]. Furthermore, MRP4 regulates platelet function through the sequestration and secretion of cyclic nucleotides as well as of certain lipid mediators including the immunomodulatory sphingosine‐1‐phosphate (S1P) [10, 11, 12, 13, 14, 15]. MRP4 may thus represent a target to interfere with platelet activation and paracrine actions. In addition, MRP4 has been shown to be involved in cell proliferation and tumorigenesis of various tumor types, for example, acute myeloid leukemia and lung cancer [16, 17, 18], and has therefore also been suggested as a target in tumor therapy. Moreover, MRP4 is involved in inflammatory processes and pain perception [19], because, in addition to S1P, it transports eicosanoids including prostaglandin E2 (PGE2) [20, 21]. In this context, a deeper understanding of the transcriptional regulation of *MRP4* is important. MRP4 overexpression in platelets, which has been linked to platelet hyperreactivity, has been observed in aspirin‐treated coronary artery disease patients [22, 23]. In addition, elevated platelet MRP4 levels have been detected in subjects infected with the human immunodeficiency virus (HIV) [24] and, more recently, in patients who have recovered from a mild course of coronavirus disease 2019 (COVID‐19) [25]. In hepatocytes, where MRP4 can be induced, for example, under cholestasis [26, 27], some xenobiotic‐inducible transcription factors have been shown to affect MRP4 levels, including the NF‐E2‐related factor 2 (Nrf2), the constitutive androstane receptor (CAR), and the peroxisome proliferator‐activated receptor α (PPAR‐α) [28, 29, 30]. In tumor cells, the proto‐oncogene MYC seems to play not only a role in regulating *MRP4* expression [31, 32] but also a role for Sp1 has been suggested [33].

Although various conditions and factors that affect *MRP4* expression have been described, transcription factors that drive the basal expression of *MRP4* have not been systematically evaluated. Here, we analyzed the activity of a series of 5′‐deletion constructs of the proximal promoter of the *MRP4* gene to identify the most crucial region for basal expression. We then characterized the binding and role of transcription factors with potential binding sites in this region, with a particular focus on Sp1.

## Methods

### Generation of luciferase reporter constructs

The reporter constructs were kindly provided by Susan P. C. Cole (Queen's University, Kingston, Canada). Briefly, a 1706‐bp fragment containing the 5′‐flanking and untranslated regions of *MRP4* were isolated from two bacterial artificial chromosome clones (Centre of Applied Genomics, Hospital for Sick Children, Toronto, Canada) and six serially deleted *MRP4* promoter reporter constructs were generated by PCR and cloned into the pGL3‐basic vector (Promega, Madison, WI, USA) [34]. Recognition sites for the restriction enzymes *Mlu*II and *Bgl*II were added to the fragments for cloning. Mutations in the putative binding sites of transcription factors were introduced by site‐directed mutagenesis using the Quick‐change II XL Mutagenesis Kit (Agilent Technologies, Santa Clara, CA, USA) under the following reaction conditions: 95 °C for 1 min, followed by 18 cycles of 95 °C for 50 s, 60 °C for 50 s, and 68 °C for 8 min, and a final elongation at 68 °C for 10 min. XL‐10 Gold Supercomp. Cells (Agilent Technologies) were used for heat shock transformation. Alternatively, the KOD Hot Start DNA Polymerase (Merck KGaA, Darmstadt, Germany) and electroporation of One Shot™ TOP10 Electrocomp™ E. coli (Thermo Fisher Scientific, Waltham, USA) cells were used for some mutations under the following reaction conditions: 95 °C for 3 min, followed by 19 cycles of 95 °C for 30 s, 65 °C for 30 s, and 72 °C for 4 min. For primer sequences, see Table 1.

**Table 1:** Nucleotide sequences of primers, probes, and siRNA.

		Sequence 5′ → 3′^a^
Primers used for mutation (MT) of Sp1 and Ets1 binding sites in the reporter constructs		
MRP4_1xSp1_MT_for	forward	CAC GCCCCGTCC **TT** GCCTCTC AGGC
MRP4_1xSp1_MT_rev	reverse	GCCTGA GAGGC **AA** GGACGGGGC GTG
MRP4_3xSp1_MT_for	forward	CGGGCGCCGGCGGCAC GCC **TT** GTCC **TT** G **TT** TCTC AGGCCCGCCGCCTC
MRP4_3xSp1_MT_rev	reverse	GAGGCGGCGGGCCTGA GA **AA** C **AA** GGAC **AA** GGC GTGCCGCCGGCGCCCG
MRP4_3xSp1_short_f^b^	forward	CGGGCGCCGGCGGCAC GCC **TT** GTCC **TT** G **TT** TCTC AACGCGTAAGAGCT
MRP4_3xSp1_short_r^b^	reverse	AGCTCTTACGCGTTGA GA **AA** C **AA** GGAC **AA** GGC GTGCCGCCGGCGCCCG
MRP4_Ets1_MT_for	forward	AGCCTGTGAAGCAG CCGCT **AA** CGGG AGCCGGGCGCCGGC
MRP4_Ets1_MT_rev	reverse	GCCGGCGCCCGGCT CCCG **TT** AGCGG CTGCTTCACAGGCT
Primers used for qPCR in chromatin immunoprecipitation assays		
CHIP‐MRP4‐Sp1‐for	forward	GGTACCCCTGGGCGTAGCTCTGG
CHIP‐MRP4‐Sp1‐rev	reverse	CTCGGCTGGAGCCTGTGAAGCAG
EMSA probes (Sp1 and Ets1 binding sites)		
MRP4‐Sp1_f	forward	CAC GCCCCGTCCCCGCCTC TCAGGCa
MRP4‐Sp1_r	reverse	gatctGCCTGA GAGGCGGGGACGGGGC GTgtac
MRP4‐Sp1_mut_f	forward	CAC GCCCCGTCC **TT** GCCTC TCAGGCa
MRP4‐Sp1_mut_r	reverse	gatctGCCT GAGAGGC **AA** GGACGGGGC GTgtac
MRP4‐Sp1_mut3_f	forward	CAC GCC **TT** GTCC **TT** G **TT** TC TCAGGCa
MRP4‐Sp1_mut3_r	reverse	gatctGCCT GAGA **AA** C **AA** GGAC **AA** GGC GTgtac
Sp1‐consensus_f	forward	ATTCGATCG GGGCGG GGCGAGCa
Sp1‐consensus_r	reverse	gatctGCTCGCC CCGCCC CGATCGAATgtac
MRP4‐cEts1_f	forward	GAAGCAG CCGCTTCCGGG AGCCGGGca
MRP4‐cEts1_r	reverse	gatctGCCCGGCT CCCGGAAGCGG CTGCTTCgtac
MRP4‐cEts1_mut_f	forward	GAAGCAG CCGCT **AA** CGGG AGCCGGGCa
MRP4‐cEts1_mut_r	reverse	gatctGCCCGGCT CCCG **TT** AGCGG CTGCTTCgtac
Ets1‐cons_TBX22_f	forward	GTCCAACCCCGGAAGTAGCAAGTGCCTCTC
Ets1‐cons_TBX22_r	reverse	GAGAGGCACTTGCTACTTCCGGGGTTGGAC
siRNA (anti‐Sp1 and control)		
siRNA control (anti GFP)	sence	CATACGCTCTCCATCAAAACAAAACG
siRNA Sp1 I	sence	GCCAAUAGCUACUCAACUA
siRNA Sp1 II	sence	GGGCAGACCUUUACAACUC

^a^ Mutated nucleotides are shown in boldface, and the putative transcription factor binding regions are underlined. Unspecific nucleotides in the EMSA probes are given in lowercase letters.

^b^ Primers used for mutation of the short construct (MRP4‐69).

### Cell culture, transfection and reporter gene assays

HeLa (RRID: CVCL_0030) and Caco2 (RRID: CVCL_0025) cells were obtained from the American Type Culture Collection (ATCC, Manassas, VA, USA); HEK293 cells (RRID: CVCL_0045) were from the European Collection of Authenticated Cell Cultures (ECACC, Salisbury, UK). The human megakaryoblastic leukemia cell line M07e (RRID: CVCL_2106) [35] was kindly provided by Joachim Boos, Department of Pediatric Hematology and Oncology, University Children's Hospital, Münster, Germany. The commercially obtained cell lines were supplied with authentication documentation from the vendors. Upon receipt, cells were expanded, aliquoted, and cryopreserved. For all experiments, cells were cultured only for a limited number of passages to minimize the risk of cross‐contamination and changes of the cell characteristics. In addition, cell line authentication was routinely performed using morphology check by microscope. The cells were also routinely tested for mycoplasma contamination by PCR, with no mycoplasma detected in the cells used in the experiments. Cells were grown at 37 °C and 5% CO_2_ in Dulbecco's Modified Eagle's (DMEM) (for HeLa and HEK293), RPMI‐1640 (for M07e), or IMDM/Ham's F‐12 (1 : 1) (for Caco2) medium. All media were obtained from Pan Biotech (Aidenbach, Germany) and supplemented with 10% fetal bovine serum, 1% nonessential amino acids, 100 U/mL penicillin, and 100 μg/mL streptomycin.

For reporter gene assays, cells were seeded at a density of 1.125 × 10^5^ cells/well in 24‐well plates, cultured for 48 h, and then transfected in antibiotic‐free medium with 500 ng of the reporter constructs containing the *MRP4* promoter fragments along with 10 ng of Renilla luciferase control vector pRL‐SV40 (Promega) using Lipofectamine^®^ 2000 (Thermo Fisher Scientific). The transfection medium was substituted with antibiotic‐free culture medium after 5 h. Reporter activity was determined 24 h after transfection using the Dual‐Luciferase Reporter Assay System (Promega) according to the manufacturer's instructions. Luciferase activities were measured in technical duplicates with an Infinite^®^ M200 microplate reader (Tecan Group Ltd, Männedorf, Switzerland) and firefly luciferase activities were normalized to renilla luciferase activities.

### Isolation of nuclear proteins and electrophoretic mobility shift assay (EMSA)

Nuclear proteins from HeLa and M07e cells were isolated using a modified version of the protocol outlined in the CelLytic™ NuCLEAR™ Extraction Kit (Sigma‐Aldrich, St. Louis, MO, USA) with the adjustments previously described [36]. Briefly, 1 × 10^7^ cells were harvested by centrifugation (300 × ***g***, 5 min, 4 °C) and subsequently washed twice in precooled PBS with the addition of 1 mm sodium orthovanadate (600 × ***g***, 5 min, 4 °C). The volume of the cell pellet (packed cell volume, PCV) was estimated and the pellet was resuspended in a fivefold volume of precooled buffer A (10 mm HEPES‐KOH pH 7.9 at 4 °C, 1.5 mm MgCl_2_, 10 mm KCl, 0.5 mm DTT, 2.5 mm PMSF, 1 mm sodium orthovanadate) and incubated on ice for 20–40 min. To enhance the disruption, a Dounce homogenizer was used. Subsequently, Nonidet P‐40 was added to a final concentration of 1%, the suspension was vortexed vigorously and centrifuged at 11 000 × ***g*** for 2 min at 4 °C. The supernatant was discarded and the pelleted nuclei were overlaid with 2/3× PCV of precooled buffer B (20 mm HEPES‐KOH pH 7.9 at 4 °C, 420 mm NaCl, 1.5 mm MgCl_2_, 0.2 mm EDTA, 25% glycerol, 1% Nonidet P‐40, 0.5% sodium deoxycholate, 0.5 mm DTT, 2.5 mm PMSF, and 1 mm sodium orthovanadate), shaken at 1800 rpm (30 min, 4 °C), and then centrifuged at 17 000 × ***g*** (10 min, 4 °C). The extraction step was repeated with 1/3× PCV and agitation for 3 h. The protein concentration of the pooled supernatants containing the nuclear proteins was determined using the Pierce™ BCA Protein Assay Kit (Thermo Fisher Scientific).

For the EMSAs, IRDye^®^700‐labeled oligonucleotide probes were purchased from Metabion International AG (Planegg, Germany) (sequences are given in Table 1). For binding reactions, 20 μg nuclear proteins was pre‐incubated for 10 min on ice in a 19‐μl reaction mixture containing 20 mm HEPES, pH 7.8, 1 mm EDTA, 0.5 mm DTT, 140 mm KCl, 10% glycerol, and 2 μg of the nonspecific competitor poly(dI‐dC). Subsequently, 50 fmol of the labeled probe was added and the mixture was then incubated on ice for 15 min. In cold competition assays, 20‐ and 200‐fold excesses of unlabeled competitor probes were used. Supershift assays were performed with 2 μg of a monoclonal anti‐Sp1 (1C6) antibody and control IgG (Santa Cruz Biotechnology, Dallas, TX, USA). Competitor probes and antibodies were added prior to addition of the labeled probe and incubated on ice for 10 min and 1 h, respectively. The binding reactions were mixed with loading buffer (26% v/v glycerol, 0.5% w/v Orange G) and resolved on a 4% (EMSA with supershift) or 5% (EMSA without supershift) native polyacrylamide gel. Signals were detected using an Odyssey^®^ CLx Imaging System (LI‐COR, Lincoln, NE, USA) with excitation wavelength of 700 nm.

### Chromatin immunoprecipitation and qPCR

Chromatin immunoprecipitation assays were performed essentially according to the protocol established by Gade and Kalvakolanu [37]. HeLa cells (2–3 × 10^7^) were grown to confluence and then exposed to a 0.5% paraformaldehyde solution (10 min, room temperature). The cross‐linking reaction was quenched by the addition of glycine to a final concentration of 0.125 m. After a washing step with PBS, cells were collected, pelleted at 2000 × ***g*** (10 min, 4 °C) and lysed on ice in hypotonic buffer containing 10 mm HEPES (pH 7.5), 10 mm KCl, 0.1 mm EDTA, 0.1 mm EGTA, 1 mm DTT and protease inhibitors (0.1 mm PMSF, 3 μg/mL aprotinin, 0.5 μg/mL leupeptin) for 20 min. Nonidet P‐40 was added to a final concentration of 0.02%, followed by incubation on ice for 10 min. The cell suspension was sheared using a 24G needle and the nuclei were pelleted by centrifugation at 13 000 × ***g*** (15 min, 4 °C) and resuspended in 50 mm Tris–HCl (pH 8.0) supplemented with 5 mm CaCl_2_ and protease inhibitors. Fragmentation of the chromatin was achieved by digestion with micrococcal nuclease (Thermo Fisher Scientific; 20 U/ 500 μL) at 37 °C for 5 min and the reaction stopped by adding EGTA (pH 8.0) to a final concentration of 20 mm. An aliquot of the digest was taken as an input control. The input DNA was reverse cross‐linked and purified as described below. To immunoprecipitate the transcription factor‐bound DNA fragments, the chromatin suspension was diluted in 20 mm Tris–HCl (pH 8.0), 150 mm NaCl, 2 mm EDTA supplemented with 1% Trition X‐100 and protease inhibitors (final volume 1.5 mL) and first precleared by incubation with washed protein A/G PLUS agarose beads (Santa Cruz Biotechnology) for 1 h with rotation at 4 °C, followed by centrifugation (500 g, 1 min) to remove the beads. Protein A/G agarose beads (25 μL) were blocked by incubation with herring sperm DNA (Promega; 75 ng/μl beads) and then added to the precleared chromatin suspension together with either 5 μg of a purified rabbit polyclonal anti‐Sp1 IgG (Merck KGaA) or a control rabbit IgG isotype control. After incubation at 4 °C overnight with rotation, beads were washed once each with low salt buffer (150 mm NaCl, 0.1% SDS, 1%Triton X‐100, 2 mm EDTA, 20 mm Tris–HCl, pH 8.1), high salt buffer (500 mm NaCl, 0.1% SDS, 1%Triton X‐100, 2 mm EDTA, 20 mm Tris–HCl, pH 8.1), LiCl buffer (0.25 m LiCl, 1% IGEPAL‐CA630, 1% deoxycholic acid, 1 mm EDTA, 10 mm Tris–HCl, pH 8.1) and TE buffer (10 mm Tris–HCl, 1 mm EDTA, pH 8.0). For each washing step, beads were incubated with the buffer (1 mL, 5 min) with rotation, followed by centrifugation (500 × ***g***, 1 min) and removal of the supernatant. For elution of the immune complexes and reversal of the DNA‐protein crosslinking,100 μL of 0.1 m NaHCO_3_ with 1% SDS and 1 μL proteinase K solution (Thermo Fisher Scientific; 20 mg/mL) were added. The samples were incubated for 2 h at 62 °C, followed by 10 min at 95 °C. Subsequently DNA was extracted using the NucleoSpin^®^ Tissue kit (Macherey‐Nagel GmbH, Düren, Germany).

The immunoprecipitated DNA fragments and the input DNA were analyzed by qPCR using primers specific for the putative Sp1 binding region in the *MRP4* proximal promoter (Table 1) and the PowerUp^®^SYBR^®^ Green Master Mix (Thermo Fisher Scientific). Due to the high GC content of the region, Q‐Solution (QIAGEN GmbH, Hilden, Germany) was added to optimize the PCR conditions. Thermal cycling conditions were as follows: 50 °C for 2 min, 95 °C for 10 min, 40 cycles of 95 °C for 15 s and 68 °C for 1 min. A standard curve was generated using the pGL3‐MRP4‐1706 promoter construct in a ten‐fold serial dilution to evaluate the efficiency and linearity of the assay.

### siRNA transfection and western blot

To knockdown Sp1 by RNA interference, a combination of two siRNAs was used, which had previously been described [38, 39] (sequences are given in Table 1). The knockdown efficiency of the siRNAs was initially evaluated through immunofluorescence staining of Sp1 after transfection in comparison to a control siRNA (anti‐GFP). For siRNA transfection with subsequent protein analysis by western blot, HeLa cells were seeded in a 6‐well plate (2 × 10^5^ cells/well) and cultured to 80–90% confluence. The siRNA was preincubated with transfection reagent (Lipofectamine^®^ RNAiMAX) in OptiMEM^®^ medium (both Thermo Fisher Scientific) (75 pmol siRNA and 7.5 μL reagent/well) and added to the cells in antibiotic‐free culture medium. The transfection medium was replaced after 40 h and cells were harvested for western blot analysis 60 h after transfection. Cells were lysed in RIPA buffer (150 mm NaCl, 0.1% sodium dodecyl sulfate, 0.5% sodium deoxycholate, 50 mm Tris–HCl, pH 8, 1% Triton X‐100) containing protease inhibitors and incubated on ice for 45 min with regular vortexing and one sonication step after 30 min. Proteins (30 μg/lane) were incubated in SDS sample buffer at 37 °C for 20 min, separated on an 8% polyacrylamide gel and transferred to nitrocellulose membranes as described [25]. After blocking in protein‐free blocking buffer (Intercept^®^, LI‐COR Biosciences GmbH), membranes were incubated with the rat anti‐MRP4 monoclonal antibody (M4‐I‐10, Enzo Life Sciences, Lörrach, Germany, 1 : 1000) and the rabbit polyclonal anti‐Sp1 IgG (Merck KGaA, 1 : 1000). β‐actin was stained as a loading control (ACTBD11B7 antibody; Santa Cruz Biotechnology; 1 : 1000). IRDye^®^ fluorescence labeled secondary antibodies (LI‐COR Biosciences GmbH) were used for detection.

### Substrate accumulation assay

To assess MRP4 function after Sp1 knockdown, HeLa cells were cultured in 24‐well plates and transfected with anti‐Sp1 or control siRNA as described above, with amounts and volumes adjusted for the 24‐well format. Accumulation assays were performed 60 h after transfection. Cells were washed with incubation buffer (142 mm NaCl, 5 mm KCl, 1 mm KH_2_PO_4_, 1.5 mm CaCl_2_, 1.2 mm MgSO_4_, 5 mm glucose, 12.5 mm HEPES, pH 7.3) and pre‐incubated with the specific MRP4 inhibitor Ceefourin‐1 [40] (50 μm) or the respective amount of the solvent (DMSO, final concentration 0.5%) in incubation buffer for 15 min at 37 °C. Subsequently, the bis‐pivaloyloxymethyl ester of [^3^H] 9‐(2‐phosphonylmethoxyethyl)adenine (bis‐POM‐[^3^H]PMEA, also known as [^3^H]Adefovir dipivoxil) (Hartmann Analytics, Braunschweig, Germany) was added to a final concentration of 150 nm, and cells were incubated at 37 °C for a further 60 min. Accumulation of [^3^H]PMEA was stopped by removing the substrate and washing the cells four times with ice‐cold PBS. The cells were lysed with 250 μL/well of an aqueous solution containing 5 mm EDTA and 0.2% SDS. An aliquot of the lysate was mixed with scintillation cocktail (Rotiszint^®^ Eco Plus^®^, Carl Roth, Karlsruhe, Germany), and radioactivity was determined in a scintillation counter. The data were normalized to the protein concentration in each well.

### Statistical analyses

Data are presented as mean ± standard error of the mean (SEM) or standard deviation (SD), and analyzed by one‐way analysis of variance (ANOVA) or unpaired *t‐*test as detailed in the figure legends (graphpad Prism 5.01 software, graphpad, SanDiego, CA, USA). *P* values < 0.05 were considered significant.

## Results

### The −69/−17 region of the *MRP4* promoter is crucial for basal transcription

**Fig. 1:**
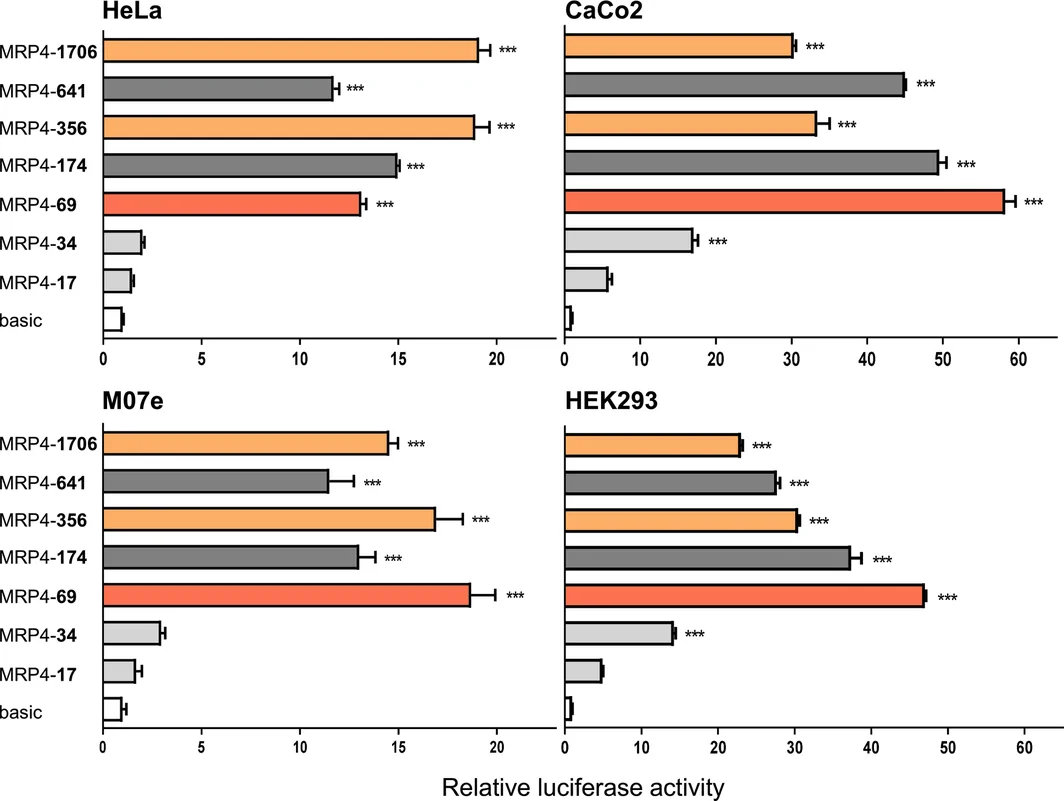
Activity of *MRP4* proximal promoter fragments in four human cell lines derived from different tissues. HeLa (cervical carcinoma), M07e (megakaryoblastic leukemia), HEK293 (embryonic kidney), and CaCo2 (colorectal carcinoma) cells (panels labeled accordingly) were transfected with a luciferase reporter vector (pGL3‐basic) containing various fragments of the *MRP4* proximal promoter, extending from the translation start site (+119) up to position −1706. Firefly luciferase activities were normalized to the activity of co‐transfected renilla luciferase and related to the activity of the empty vector (basic) (means + SEM, *n* = 9–18, at least 3 separate experiments; ***P < 0.001 significant difference compared to basic and to MRP4‐17; 1 way‐ANOVA).

To identify the region of the *MRP4* proximal promoter that is most important for basal transcriptional activity, a series of *MRP4* promoter fragments of different lengths upstream of the translation start site (+119) was used in luciferase reporter gene assays. The promoter constructs were transfected into several human cell lines derived from different tissues. These included HeLa cells, as a well‐established human tumor cell model, the megakaryoblastic leukemia cell line M07e, as a model for megakaryocytes and indirectly for platelets, as well as human embryonic kidney (HEK293) and epithelial colorectal carcinoma (CaCo2) cells, since MRP4 is also expressed in kidney and intestine (Fig. 1). In all cell lines studied, the basal promoter activity increased slightly with the −34/+119 construct, whereas it was markedly increased with the −69/+119 construct (fold increase relative to the shortest construct −17/+119: HeLa 8.9, M07e 11.0, HEK293 9.6, CaCo2 10.0). These results indicate that across all tested cell models, the −69/−17 region of the *MRP4* promoter is crucial for basal transcription. Whereas in HeLa cells promoter activity was slightly increased further when the −356/+119 and − 1706/+119 constructs were tested in the luciferase reporter assay, the −69/+119 construct had the highest activity in M07e, HEK293 and CaCo2 cells (activities normalized to −17/+119 for the −356/+119 construct: HeLa 12.8, M07e 10.0, HEK293 6.2, CaCo2 5.7; for the −1706/+119 construct: HeLa 12.9, M07e 8.6, HEK‐293 4.5, CaCo2 5.2). For subsequent analyses, we focused on the well‐characterized HeLa cell line and the megakaryoblastic M07e cells.

### The putative binding sites for Sp1 and for Ets transcription factors in this region have a decisive impact on the promoter activity

**Fig. 2:**
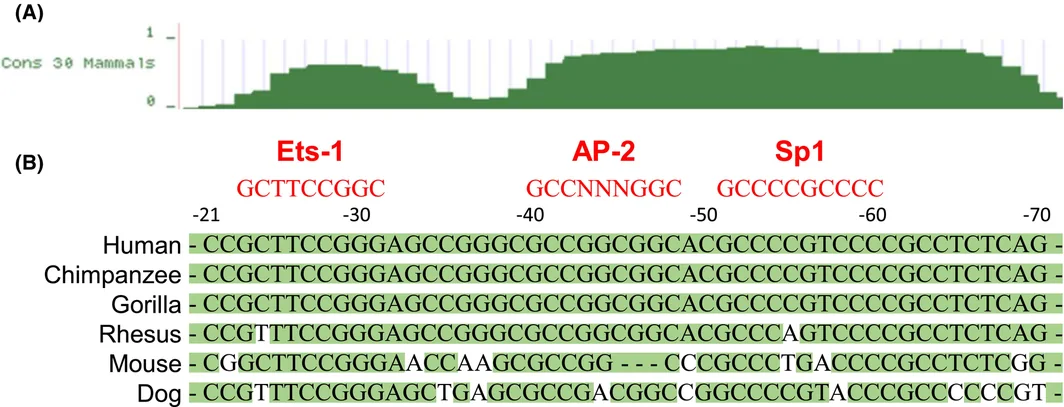
*In silico* analyses of evolutionary conservation of the proximal *MRP4* promoter. (A) Evolutionary conservation of the 5′‐proximal region of the *MRP4* gene in mammals was analyzed and illustrated using the sequence alignment and visualization tools of the UCSC Browser (https://genome.ucsc.edu/) indicating a highly conserved sequence of approximately 50‐bp upstream from position −20 of the human sequence. The Y‐axis represents the degree of conservation between the human genome and the corresponding mammalian genomes (basewise conservation across 30 mammalian species). (B) Alignment of six exemplary selected mammalian sequences. Conserved bases are highlighted in green. The consensus binding sequences for Ets‐1 (and related factors), AP‐2, and Sp1 are shown (in red) to indicate potential binding sites. For the human sequence, nucleotide positions upstream of the transcription start site are given above the alignment.

**Fig. 3:**
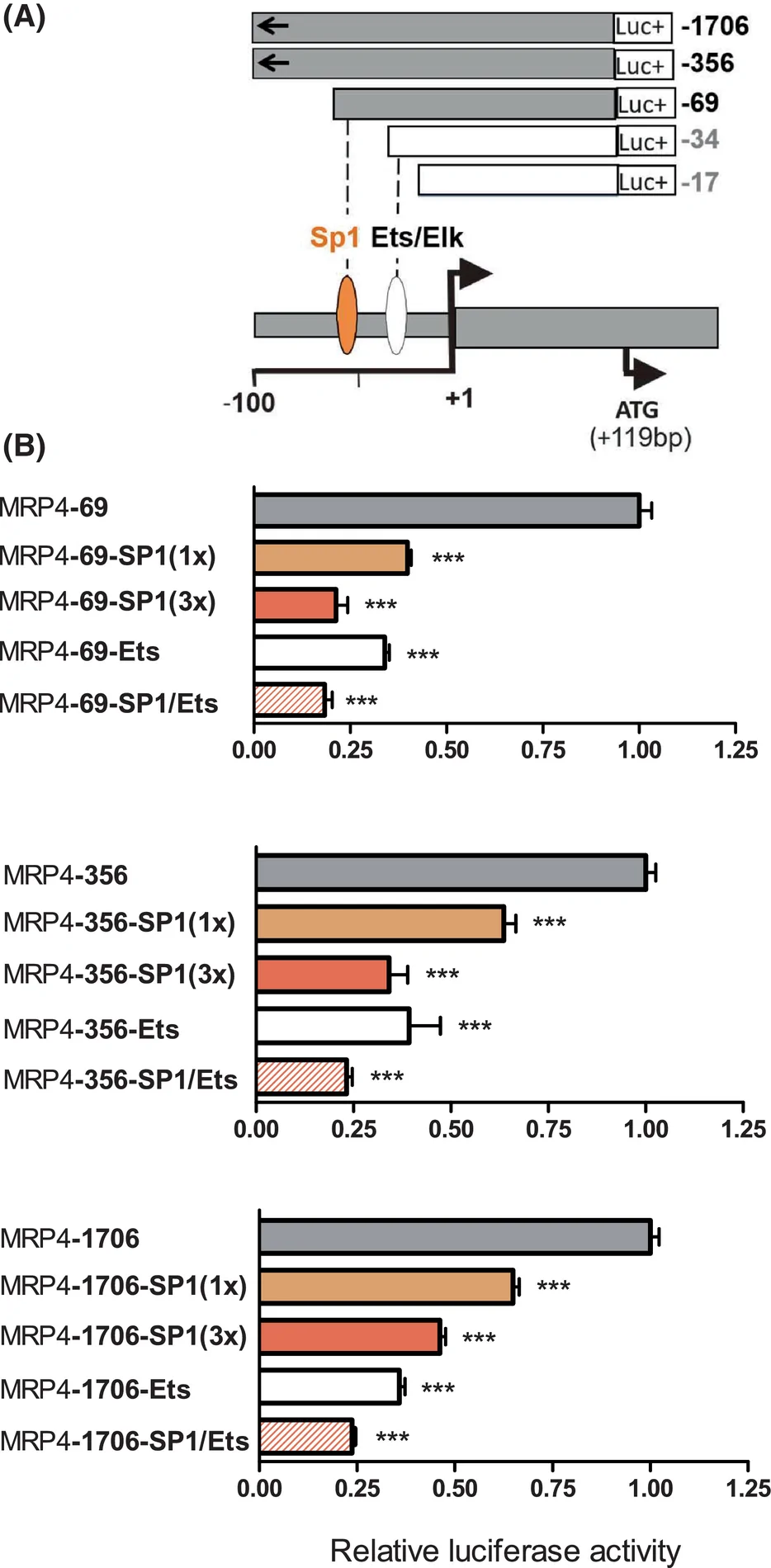
*MRP4* promoter activity in HeLa cells after mutation of the binding motifs of Sp1 and Ets factors. A) Position of the putative binding motifs of Sp1 (−64/−51) and Ets factors (−31/−23) in the promoter deletion constructs reaching from the translation start site (+119) to position −1706 in the longest fragment. B) Relative *MRP4* promoter activity (normalized to the non‐mutated fragment) in three promoter constructs of different lengths (−69/+119, ‘MRP4‐69’, upper panel; −356/+119, ‘MRP4‐356’ middle panel; −1706/+119, ‘MRP4‐1706’, lower panel) with single or triple mutated Sp1 binding site (‐Sp1(1×);‐Sp1(3×)), mutated Ets site (−Ets), or mutated Sp1(3x) and Ets sites (‐Sp1/Ets). Sequences of the mutagenesis primers are given in Table 1. (Means + SEM; *n* = 9–15, at least three separate experiments; ***P < 0.001, significant difference compared to the nonmutated fragment, 1 way‐ANOVA).

The region of about 50‐bp upstream from position −20 of the *MRP4* promoter is highly conserved across many species and contains putative binding sites for the transcription factors Sp1 (−64/−51) and for Ets‐1 and Elk‐1 or related Ets factors (−31/−23) (Fig. 2). In addition, there is also a predicted binding motif for AP‐2 between these two sequences. Site‐directed mutagenesis introducing changes in the Sp1‐binding motif in three promoter reporter constructs of different lengths led to a marked decrease in luciferase activity, indicating that Sp1 binding at this position has a major impact on the *MRP4* promoter activity. Figure 3 shows the effect of these mutations in HeLa cells. The putative Sp1 binding sequence was first mutated at a single position and subsequently at two additional positions (Table 1), since the sequence provides more than one possible binding positions. Both mutated constructs showed a significant reduced promoter activity with a stronger effect observed when three mutations were introduced. The effect was more pronounced with the shortest fragment (MRP4‐69) but was still significant in the longest construct (MRP4‐1706). We also mutated the putative Ets‐1/Elk‐1 binding site (Table 1) which also led to a decrease in the activity across all three promoter constructs. The mutation of both sites led to the most effective reduction in promoter activity. Similar outcomes were obtained in M07e cells. Here, the relative activities compared to the unmutated construct for the MRP4‐69 fragment were 0.35 ± 0.02‐fold, 0.26 ± 0.01‐fold, and 0.23 ± 0.02‐fold for the Sp1 (1×), Sp1 (3), and Ets‐1/Elk‐1 mutations, respectively (means ± SEM, *n* = 6–9). In contrast, mutation of the potential AP‐2 binding site did not reduce the promoter activity but rather led to a slight increase (1.38 ± 0.02‐fold and 1.36 ± 0.09‐fold activity of the MRP4‐69 fragment in HeLa and M07e cells, respectively; means ± SEM, *n* = 6–8).

### Sp1 and factors recognizing the Ets‐1/Elk‐1 motif bind to the identified *MRP4* promoter region

**Fig. 4:**
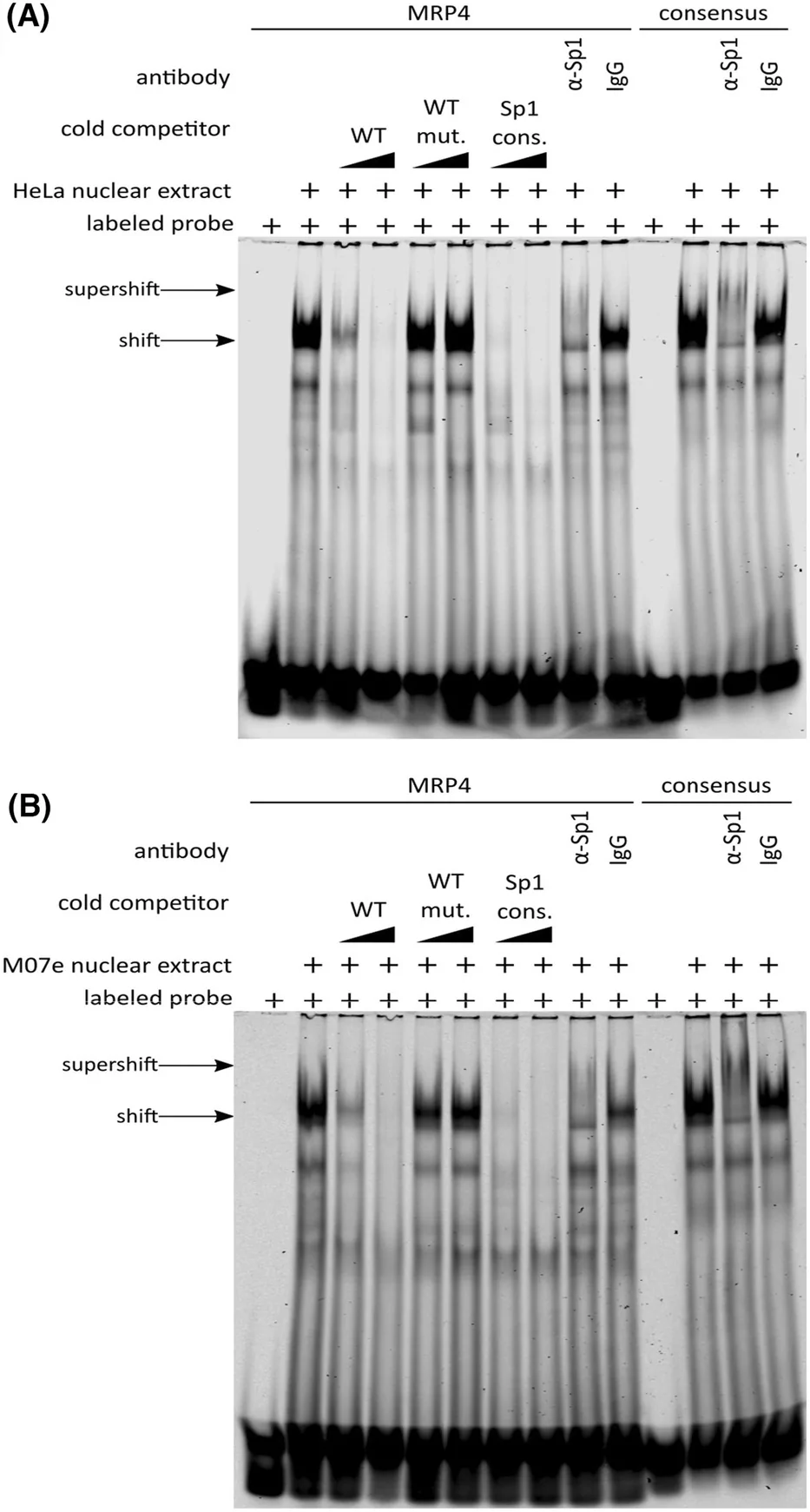
Representative EMSAs indicating *in vitro* binding of Sp1 to the *MRP4* proximal promoter. Oligonucleotides corresponding to the −47 to −72 region of the *MRP4* promoter were end‐labeled with IRDye®‐700 and incubated with nuclear extracts of HeLa (A) and M07e (B) cells. For competition assays, the unlabeled wild‐type (WT) or mutated (WT mut.) *MRP4* promoter oligonucleotides or oligonucleotides corresponding to the Sp1 consensus sequence (Sp1 cons.) were used in increasing molar excess (20–200×) (see Table 1 for sequences). The arrows indicate the shift of the bands and the supershift when an anti‐Sp1 antibody (α‐Sp1) was included in the EMSA. The mutant DNA competitor probes and the control IgG (IgG) had no effect. For comparison the labeled consensus sequences (consensus) were also used.

**Fig. 5:**
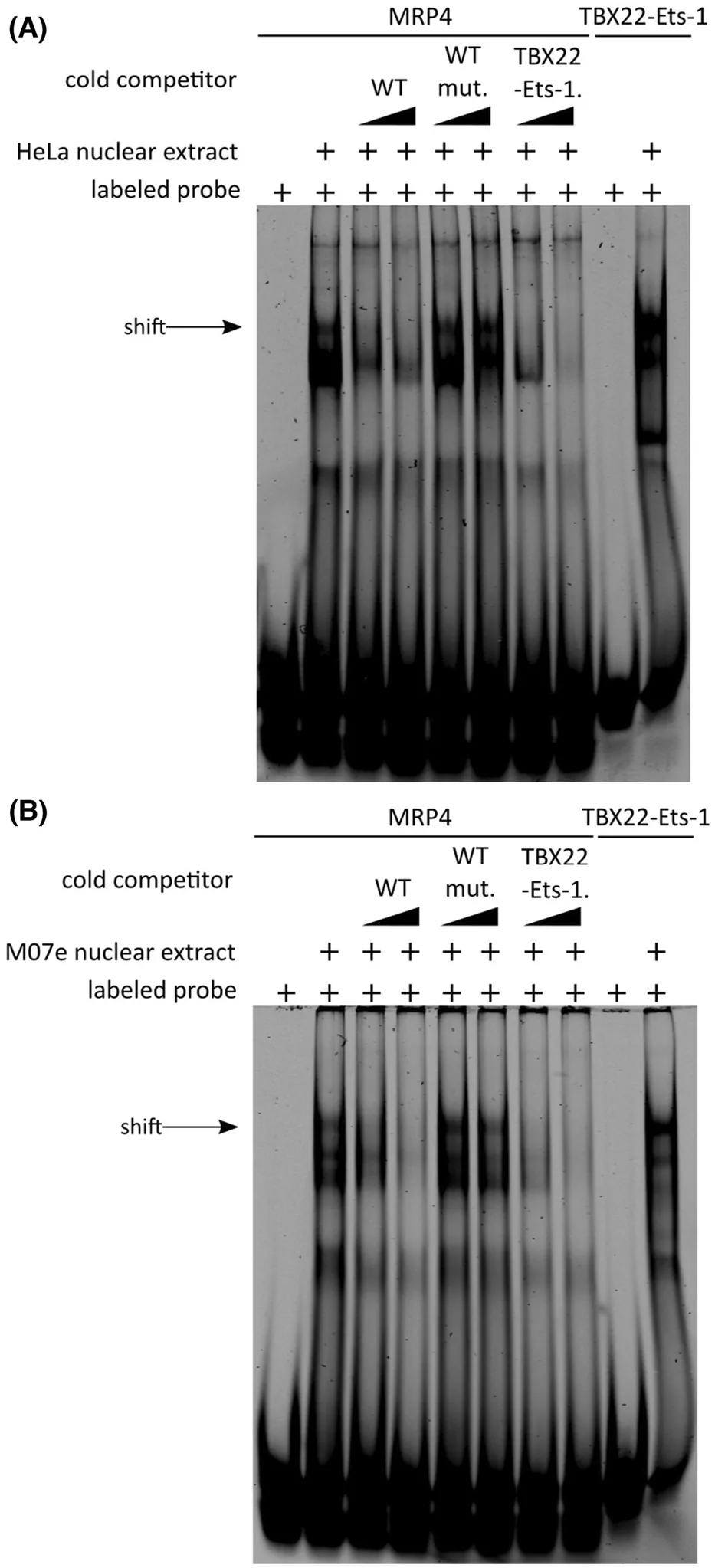
Representative EMSAs indicating the binding of Ets transcription factors to the *MRP4* promoter. Oligonucleotides corresponding to the *MRP4* promoter region and containing the recognition sequence for Ets‐1 and related factors were end‐labeled with IRDye®‐700 and incubated with nuclear extracts of HeLa (A) and M07e (B) cells. For competition assays, the unlabeled wild‐type (WT) or mutated (WT mut.) *MRP4* promoter oligonucleotides were used in increasing molar excess (20–200×). The Ets‐1 binding sequence from the promoter region of the T‐box transcription factor TBX22 (TBX22‐Ets‐1) was also used for competition assays and as a labeled positive control. The arrows indicate the shift of the bands. For sequences, see Table 1.

In order to ascertain the binding of transcription factors to these sequence segments, electrophoretic mobility shift assays (EMSA) were performed. The EMSA is based on the principle that a DNA‐protein complex will have a different electrophoretic mobility than nonbound DNA. The shifts in DNA mobility were visualized on a native acrylamide gel using labeled oligonucleotides (Figs 4 and 5). When nuclear extracts of HeLa (Fig. 4A) and M07e (Fig. 4B) cells were incubated with the labeled probes containing the predicted Sp1 binding site (−64/−51), a clear retardation of the signal was observed. Competition with the unlabeled probe in increasing excess as well as with a Sp1 consensus sequence resulted in a reduced or absent signal. In contrast, competition with an oligonucleotide corresponding to the (3×) mutated Sp1 binding site elicited no effect, thereby confirming the specificity of the Sp1 binding. When the nuclear extracts were pre‐incubated with a specific antibody against Sp1, the signal was additionally shifted (supershifted), while incubation with the control IgG had no effect.

EMSAs were also performed with the region containing the recognition sequence for Ets‐1/Elk‐1 (Fig. 5). Here, a known Ets‐1 binding sequence from the promoter region of the T‐box transcription factor TBX22 [41] was used as a positive control. An antibody‐mediated supershift was not demonstrated in this case, since not just one protein can recognize this common motif. Assays with probes containing the putative AP2 binding site did not cause any shift indicating no protein binding (data not shown).

**Fig. 6:**
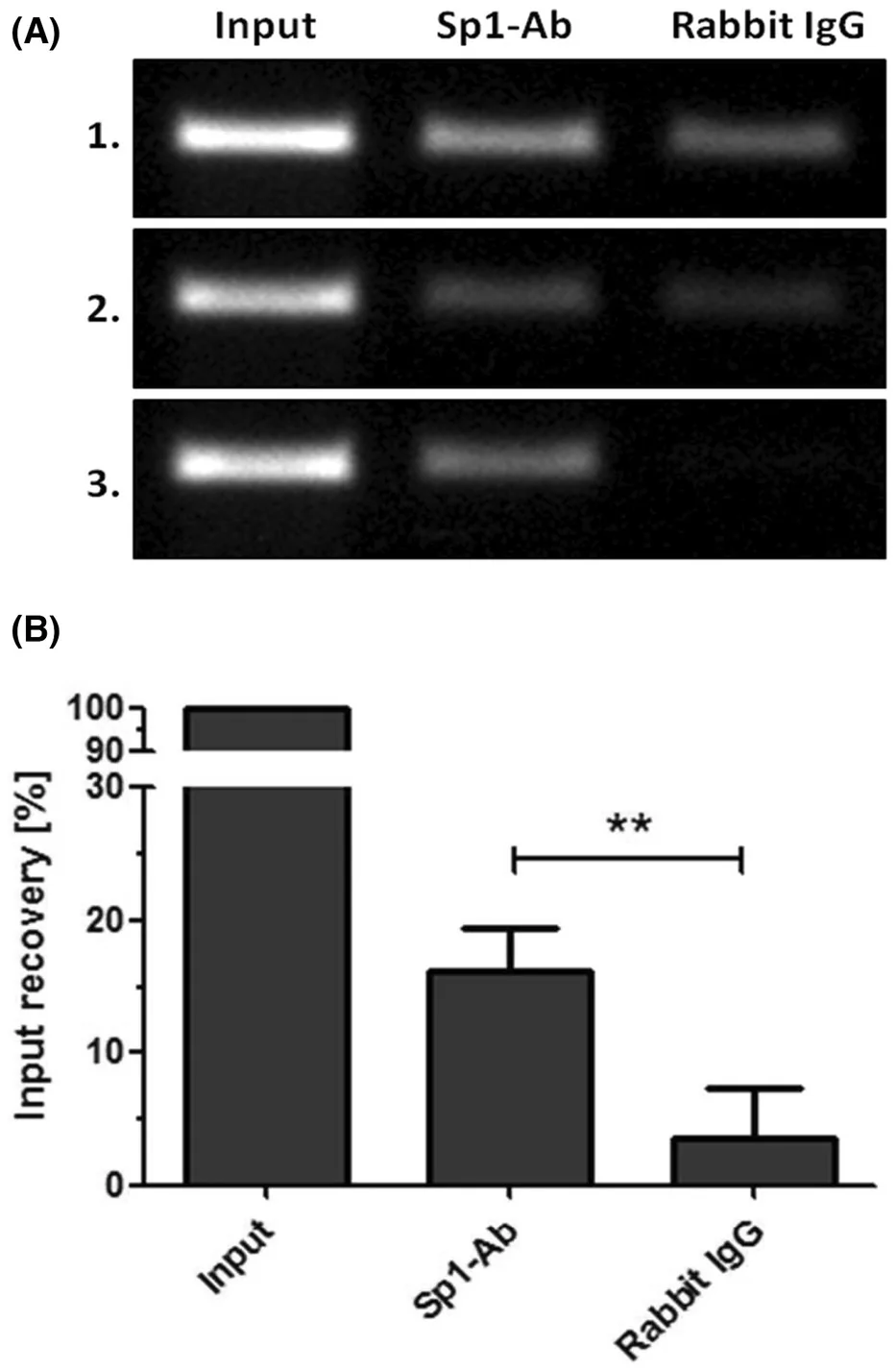
ChIP assays indicating the binding of Sp1 to the *MRP4* promoter in living cells. Proteins that interact with the DNA in the chromatin of HeLa cells were cross‐linked to the DNA by formaldehyde fixation. The chromatin was isolated and fragmented by micrococcus nuclease digestion. Anti‐Sp1 antibody was then used to capture the protein‐DNA complexes using agarose beads linked to protein A and G. The DNA was released by reverse crosslinking and analyzed by qPCR with primers specific for the putative Sp1 binding region in the *MRP4* promoter. (A) Shown are agarose gels with the amplified DNA from three independent ChIP experiments. For visualization of the results, the Sp1 binding site was amplified in a qPCR reaction (only 27 cycles) and the products were separated on a 2% agarose gel. (B) qPCR‐based signal quantification. The *MRP4* promoter DNA in the samples from each ChIP assay was quantified in duplicate by qPCR and normalized to the DNA input (means + SD, *n* = 3 separate experiments; **P < 0.01; 1‐way‐ANOVA).

The binding of Sp1 to the *MRP4* promoter region was further corroborated using a chromatin immunoprecipitation (ChIP) assay, which allows analysis of protein‐DNA interactions in the context of a living cell [37]. Thus, DNA‐protein complexes are fixed, the chromatin fragmented, and then the factor of interest is immunoprecipitated and the co‐precipitated DNA analyzed. The respective putative Sp1 binding site (−64/−51) of the *MRP4* promoter in the obtained DNA was quantified by qPCR and the input recovery was calculated from the Ct values (Fig. 6). A significant enrichment (approximately 4.5‐fold) of the *MRP4* promoter region in the precipitate was obtained with the anti‐Sp1 antibody compared to a control immunoglobulin. With the Sp1 antibody, 16.2% ± 3.2% of the input was recovered, whereas only 3.6% ± 3.7% was precipitated in the control (mean ± SD, *n* = 3).

### Knock‐down of Sp1 significantly reduces MRP4 protein levels and function

**Fig. 7:**
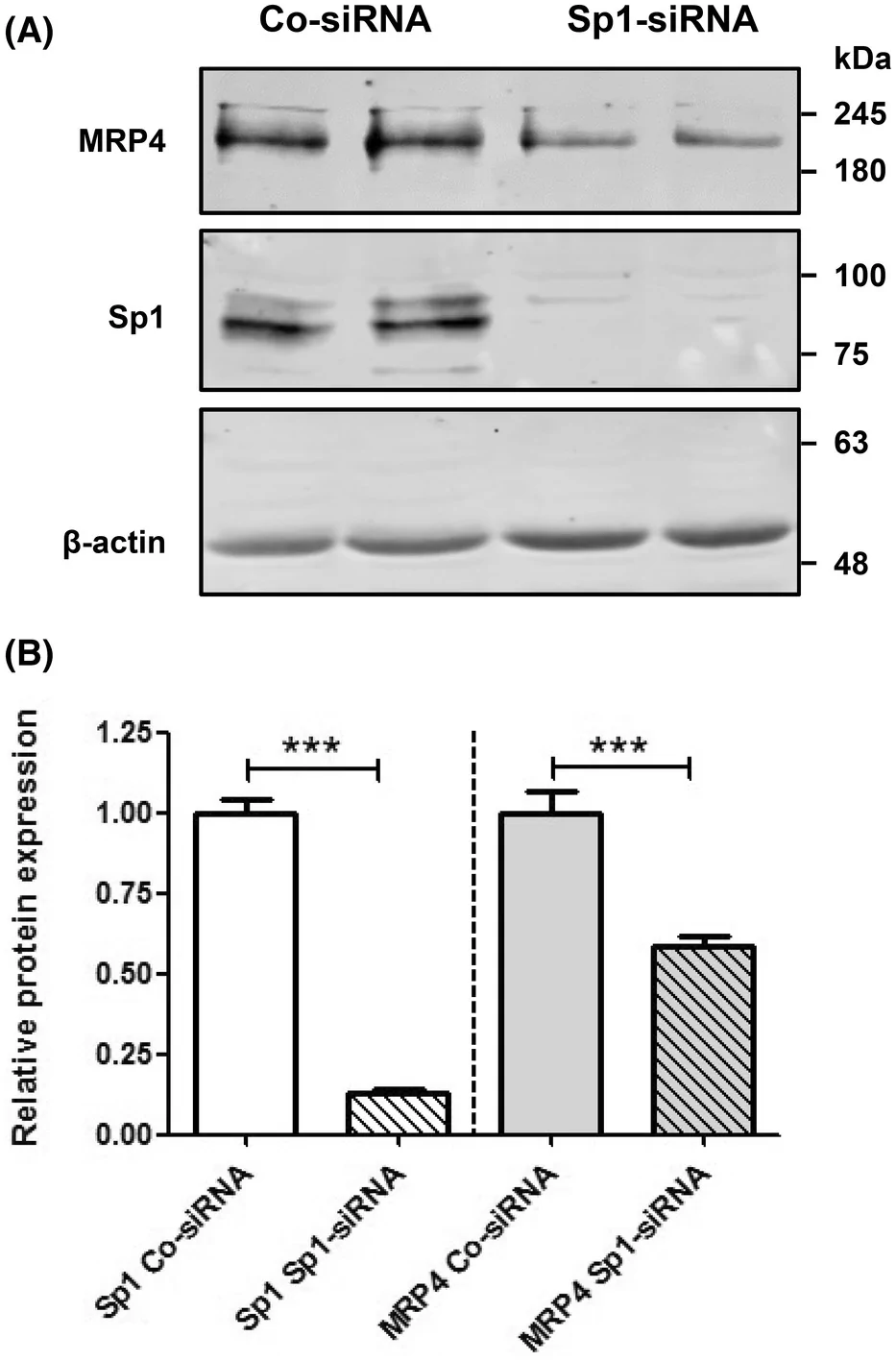
Detection of MRP4 and Sp1 proteins in HeLa cells after Sp1 siRNA knock‐down. (A) Detection of MRP4, Sp1, and β‐Actin in a representative western blot (30 μg total protein/lane) of HeLa cells 60 h after transfection. The cells transfected with Sp1‐siRNA showed almost no signal for Sp1 and the intensity of the band corresponding to MRP4 was markedly reduced compared to cells transfected with a control siRNA (Co‐siRNA). (B) Relative levels of Sp1 and MRP4 proteins. The signal intensities were normalized to the *β*‐Actin levels and expressed as percent of the mean values of the controls: For Sp1: Co‐siRNA 100 ± 4.4%; Sp1‐siRNA 12.8 ± 1.6%; for MRP4: Co‐siRNA 100 ± 6.7%; Sp1‐siRNA 58.6 ± 3.2% (means + SEM, 9 blots, 3 separate transfection experiments) (unpaired *t*‐test: ***P < 0.001).

The effect of Sp1 on MRP4 protein levels was examined using an siRNA‐based Sp1 knock‐down approach. First, the efficiency of two siRNA sequences (see methods section) was tested by measuring endogenous Sp1 levels in HeLa cells by immunofluorescence staining and western blot analyses. A combination of both siRNAs was then used, which resulted in a marked knockdown of Sp1 protein to approximately 13% of the expression levels observed in cells transfected with a control siRNA. This Sp1 knockdown was accompanied by a significantly decreased level of MRP4 protein to approximately 59% of control levels (Fig. 7).

**Fig. 8:**
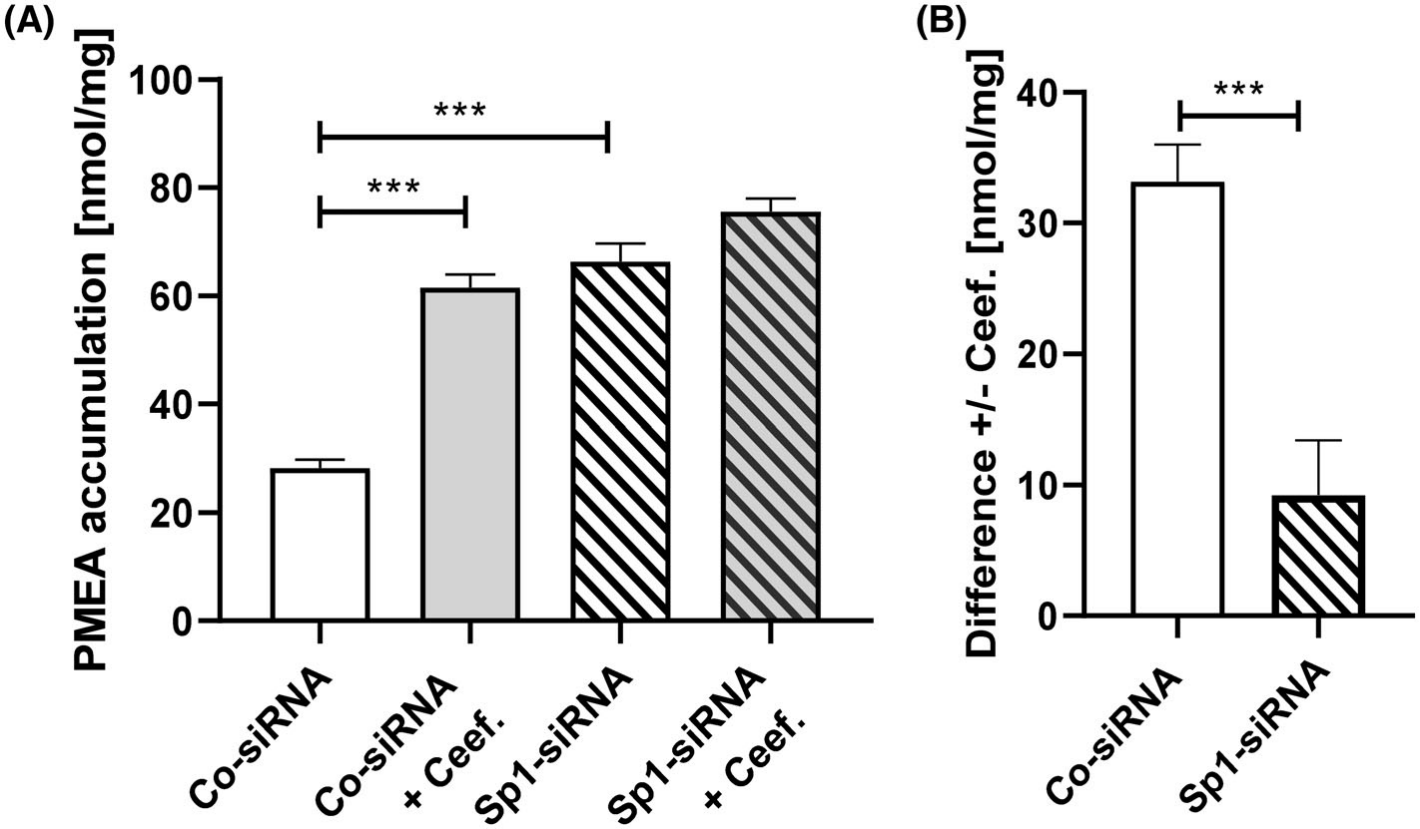
Reduced MRP4 function after Sp1 knock‐down in HeLa cells indicated by increased accumulation of the substrate Adefovir (PMEA). Accumulation assays were performed 60 h after transfection of HeLa cells with Sp1‐siRNA or control siRNA (Co‐siRNA). Cells were pre‐incubated without or with the specific MRP4 inhibitor Ceefourin‐1 (50 μm) (+ Ceef.). After adding the ester prodrug bis(POM)‐[^3^H]PMEA, the cells were incubated for 60 min at 37 °C. Subsequently, the cells were washed and lysed. The intracellular accumulation of [^3^H]PMEA, which is rapidly released from the prodrug by esterases and effluxed by MRP4, was calculated from the radioactivity detected in the lysate and normalized to the protein amount (A). The PMEA accumulation was significantly increased by MRP4 inhibition as well as after Sp1 knockdown. The increase in accumulation elicited by Ceefourin‐1 (Difference +/− Ceef.) was significantly decreased after Sp1 knockdown (B). (means + SEM, *n* = 12, 3 separate experiments; ***P < 0.001 significant difference, 1 way‐ANOVA and unpaired *t*‐test in A and B, respectively).

To assess MRP4 function after Sp1 knockdown in HeLa cells, accumulation of Adefovir (PMEA) was measured (Fig. 8). PMEA was one of the first nucleotide analogs reported to be substrates of MRP4, and its intracellular accumulation after incubation of cells with its lipophilic ester prodrug bis(POM)‐PMEA (Adefovir dipivoxil) has been established as a reliable indicator of MRP4 function [42, 43]. In addition, the effect of the specific MRP4 inhibitor Ceefourin‐1 [40] on the PMEA accumulation was measured. As shown in Fig. 8A, the intracellular PMEA accumulation was significantly increased by MRP4 inhibition as well as after Sp1 knockdown. The difference in accumulation with and without MRP4 inhibition by Ceefourin‐1, which can be regarded as a direct indicator of the substrate amount exported by MRP4, was decreased by 72% after Sp1 knockdown (Fig. 8B). These results confirm a decrease of MRP4 after Sp1 knockdown also on the functional level.

## Discussion

MRP4 has been demonstrated to play a significant physiological and pathophysiological role in both the cardiovascular and oncological fields, due to its broad tissue distribution and wide substrate spectrum, which includes a number of endogenous signaling molecules [3, 7, 44].

MRP4 is highly expressed in platelets and affects various signaling pathways by transporting not only cyclic nucleotides but also lipid mediators such as thromboxane and S1P. Inhibiting MRP4 reduces platelet aggregation and thrombus formation, in part by increasing cytosolic cAMP/cGMP and decreasing the export of thromboxane, thus impairing platelet activation and thrombus formation [10, 11, 12, 13, 14, 15]. MRP4 has also been detected in vascular smooth muscle and endothelial cells, where its regulation of cyclic nucleotides may influence vascular tone, remodeling, and responses to injury [5, 7]. In addition, it is expressed in cardiomyocytes, where it has been shown to impact cardiac contractility, hypertrophy, and overall cardiac function [6, 7]. Lastly, MRP4 overexpression has been associated with platelet hyperactivity in a variety of contexts, including systemic inflammation following viral infection [24, 25].

MRP4 also plays a significant role in oncology. In this context, it is relevant to resistance to chemotherapeutic agents, such as thiopurines and methotrexate, but also affects tumor biology in a drug‐independent manner [16, 18]. Elevated MRP4 levels have been observed in several tumor types including acute leukemia [16, 17], neuroblastoma [45], pancreatic [9, 46], lung [18, 47], colorectal [48], prostate [49, 50], breast [51], and ovarian cancers [33, 52]. *MRP4* overexpression has often been associated with increased cell proliferation, decreased apoptosis and chemosensitivity, and overall poor prognosis. Moreover, MRP4 has been shown to promote an inflammatory microenvironment through the release of PGE2, which may consequently result in increased angiogenesis, migration, and immune evasion [50, 51]. It is therefore essential to acquire more knowledge about the key factors that regulate *MRP4* transcription.

In the present study, we have shown that: First, the −69/−17 promoter region of the *MRP4* gene is crucial for its basal transcription in four human cell lines derived from differing tissues, including HeLa, M07e, HEK‐293, and CaCo‐2 cells. Constructs containing this small region of the *MRP4* promoter showed a marked increase in reporter gene activity compared to shorter constructs, indicating it is the core active region for cell‐type independent basal expression. Second, this region of the proximal promoter is highly conserved and contains putative binding sites for the transcription factors Sp1 and Ets transcription factors. Mutations introduced into these sites significantly reduced the promoter activity, confirming the essential role of these sequence motifs in gene activation. Third, we have shown directly that these transcription factors bind to their respective motifs in the *MRP4* promoter, as evidenced by EMSA and (for Sp1) ChIP assays. Fourth and finally, we have shown that siRNA‐mediated knockdown of Sp1 via siRNA results in a significant decrease in MRP4 protein levels and function, indicating Sp1 exerts direct regulatory control of *MRP4* gene expression.

Sp1 is a universally expressed and highly conserved transcription factor that binds to GC‐rich DNA sequences. It controls genes that are crucial for cell proliferation, apoptosis, DNA‐damage response, and tumor progression [53, 54]. Sp1 is frequently overexpressed in a variety of tumors, and Sp1 levels have been reported to correlate with more advanced cancer stages and worse prognosis. Moreover, Sp1 has been recently identified as a critical factor in regulating vascular calcification, myocardial fibrosis, inflammation, and other pathological processes related to cardiovascular diseases [55]. Members of the Ets protein family (such as Ets‐1 and Elk‐1) represent additional transcription factors influencing gene expression related to cell growth, differentiation, and migration, as well as inflammation [56]. Their transcriptional activity is stimulated via mitogen‐activated protein kinase (MAPK) signaling pathways, especially in response to growth factors, cytokines, and oxidative stress [56]. Ets‐1 and Elk‐1 can physically interact with Sp1 [57, 58], and their binding sites are in close proximity in the proximal promoter region of *MRP4*, suggesting coordinated regulation. They may act together as a hub integrating diverse signals—such as oxidative stress, pro‐inflammatory mediators, and second messengers like cAMP—to regulate levels of MRP4. It is known that cAMP plays an important role in cell proliferation and differentiation, especially in normal as well as leukemic blood cells [59, 60]. We and others have demonstrated that MRP4 not only regulates cAMP levels but is in turn itself regulated by cAMP. The transcriptional upregulation of MRP4 by cAMP involves at least in part the EPAC/MEK pathway [8, 9] and possibly modulation of Ets‐1/Elk‐1 and Sp1. MRP4 can also be upregulated by PGE2, which raises intracellular cAMP through its receptors (especially EP2/EP4), and is itself also exported from cells by MRP4, leading to a positive feedback loop especially in the context of tumor progression and inflammation [18, 50, 51]. In addition, MRP4 has been observed to be transcriptionally upregulated by histamine, specifically through the H2 receptor, also involving cAMP/EPAC‐PKA pathways [17].

Upstream of this core region of the proximal promoter responsible for cell‐type independent basal expression are several additional putative binding sites for various transcription factors, including Nrf2, PPAR‐α, and hepatocyte nuclear factors (HNFs), which may be involved in more tissue‐specific regulation. When longer fragments of the promoter were tested in reporter assays, two of them (−356/+119 and − 1706/+119) showed slight increases in activity when introduced into HeLa cells, indicating the presence of additional positive regulatory elements. In M07e, HEK293, and CaCo‐2 cells, the shorter −69/+119 fragment had the highest activity, suggesting that in these cell types predominantly negatively regulatory factors bind to upstream sequences under basal conditions. These additional positive and negative regulatory elements in the promoter may allow for fine‐tuned, context‐dependent expression control in different cell types.

### Conclusions

Our findings underscore the importance of Sp1 and Ets factors as major regulators of *MRP4* expression, potentially integrating MRP4‐mediated export activity with various physiological signaling pathways. Understanding the mechanisms governing *MRP4* expression could facilitate modulation of MRP4 levels in cardiovascular and oncological disorders.

## Conflict of interest

The authors declare that the research was conducted in the absence of any commercial or financial relationships that could be construed as a potential conflict of interest.

## Author contributions

DS and AK performed and analyzed the main experiments (DS – CHIP assays, siRNA knockdown, AK – EMSA assays, site‐directed mutagenesis). KZ, JG, SG, and SB performed and analyzed reporter gene assays. GJ and MVT conceived and supervised the project. GJ wrote the manuscript draft and all authors have read and edited the manuscript.

## Data Availability

The original datasets underlying this article will be shared upon reasonable request from the corresponding authors. Data are located in controlled access data storage at University Medicine Greifswald.
